# Hypoxia-inducible factor 1α modulates interstitial pneumonia-mediated lung cancer progression

**DOI:** 10.1186/s12967-023-04756-6

**Published:** 2023-11-27

**Authors:** Kiyofumi Shimoji, Taku Nakashima, Takeshi Masuda, Masashi Namba, Shinjiro Sakamoto, Kakuhiro Yamaguchi, Yasushi Horimasu, Takahiro Mimae, Shintaro Miyamoto, Hiroshi Iwamoto, Kazunori Fujitaka, Hironobu Hamada, Morihito Okada, Noboru Hattori

**Affiliations:** 1 Department of Molecular and Internal Medicine, Graduate School of Biomedical and Health Sciences, Hiroshima University, 1-2-3 Kasumi, Minami-ku, Hiroshima, 734-8551 Japan; 2 Department of Surgical Oncology, Research Institute for Radiation Biology and Medicine, Hiroshima University, Hiroshima, Japan; 3 Department of Physical Analysis and Therapeutic Sciences, Graduate School of Biomedical and Health Sciences, Hiroshima University, Hiroshima, Japan

**Keywords:** Interstitial pneumonia, Lung cancer, Hypoxia-inducible factor, Alpha subunit, Tumor microenvironment, Ascorbic acid

## Abstract

**Background:**

The prognosis of patients with lung cancer accompanied by interstitial pneumonia is poorer than that of patients with lung cancer but without interstitial pneumonia. Moreover, the available therapeutic interventions for lung cancer patients with interstitial pneumonia are limited. Therefore, a new treatment strategy for these patients is required. The aim of the present study was to investigate the pathophysiological relationship between interstitial pneumonia and lung cancer and explore potential therapeutic agents.

**Methods:**

A novel hybrid murine model of lung cancer with interstitial pneumonia was established via bleomycin-induced pulmonary fibrosis followed by orthotopic lung cancer cell transplantation into the lungs. Changes in tumor progression, lung fibrosis, RNA expression, cytokine levels, and tumor microenvironment in the lung cancer with interstitial pneumonia model were investigated, and therapeutic agents were examined. Additionally, clinical data and samples from patients with lung cancer accompanied by interstitial pneumonia were analyzed to explore the potential clinical significance of the findings.

**Results:**

In the lung cancer with interstitial pneumonia model, accelerated tumor growth was observed based on an altered tumor microenvironment. RNA sequencing analysis revealed upregulation of the hypoxia-inducible factor 1 signaling pathway. These findings were consistent with those obtained for human samples. Moreover, we explored whether ascorbic acid could be an alternative treatment for lung cancer with interstitial pneumonia to avoid the disadvantages of hypoxia-inducible factor 1 inhibitors. Ascorbic acid successfully downregulated the hypoxia-inducible factor 1 signaling pathway and inhibited tumor progression and lung fibrosis.

**Conclusions:**

The hypoxia-inducible factor 1 pathway is critical in lung cancer with interstitial pneumonia and could be a therapeutic target for mitigating interstitial pneumonia-mediated lung cancer progression.

**Supplementary Information:**

The online version contains supplementary material available at 10.1186/s12967-023-04756-6.

## Background

Patients with interstitial pneumonia (IP), an intractable, chronic fibrotic lung disease, show approximately fivefold higher lung cancer complication rates than healthy individuals, and approximately 10% of patients with idiopathic IP ultimately develop lung cancer [1, 2].

In a retrospective observational study of patients with idiopathic pulmonary fibrosis (the most common subtype of IP), approximately 40% of patients with concomitant lung cancer had stage 3B or higher cancer, despite follow up with chest X-rays every 3 months and annual CTs [3]. This suggests that lung cancer with IP may progress more rapidly than lung cancer without IP.

Patients with IP often develop acute exacerbations and rapid progressive respiratory failure accompanied by new infiltrate shadows in both lungs [4]. Unfortunately, all major therapeutic strategies for lung cancer, including surgery, radiotherapy, and chemotherapy, can induce acute exacerbations in these patients [5–7], and acute IP exacerbations can still occur in some patients without apparent triggers. Despite advances in minimally invasive surgery, high-accuracy radiotherapy, and new chemotherapeutic agents (immune checkpoint and tyrosine kinase inhibitors), therapeutic interventions for lung cancer patients with IP are limited and their prognosis remains poorer than that of patients with lung cancer without IP [8].

Pharmaceutical interventions for IP using antifibrotic agents, such as pirfenidone and nintedanib, and immunosuppressants, such as steroids, have been attempted [9–11]. However, reports on the effects of antifibrotic agents on lung cancer with IP are limited. Given their suppressive effect on immune cells, immunosuppressants repress anti-cancer immunity and accelerate lung cancer progression [12]. Therefore, managing lung cancer with IP requires two simultaneous contradictory actions: exerting cytotoxic effects on cancer cells while exerting cytoprotective effects on lung component cells. This strategy has yet to be established as it requires validating the pathophysiological relationship between IP and lung cancer. It is well accepted that local inflammation and tissue damage can alter the characteristics of cancers in organs other than the lungs [13–15]; however, such reports for lung cancer are limited due to the lack of available animal models for investigating the interactions between IP and lung cancer.

In the present study, we hypothesized that IP-mediated changes in the lungs render the phenotype of lung cancer and its tumor microenvironment aggressive. To study this hypothesis, we developed a novel murine model of lung cancer with IP to investigate the effects of IP on lung cancer and the tumor microenvironment. We identified the HIF-1 signaling pathway as a critical player in the murine model of lung cancer with IP. Moreover, we aimed to propose a therapeutic strategy targeting the HIF-1 signaling pathway. Various HIF-1 inhibitors have been investigated in animal models [16]; however, specific HIF-1 inhibitors cannot be used in clinical practice due to the protective role of HIF-1 against cardiovascular disease [17]. Therefore, the clinical safety of HIF-1 inhibitors is not guaranteed. To address this limitation of existing HIF-1 inhibitors, we evaluated ascorbic acid (AsA, which is known to promote HIF-1 degradation) as a therapeutic candidate for lung cancer with IP.

## Methods

### Animals

Female C57BL/6 and B6D2F1/Crl mice (6–8 weeks old; Charles River Laboratories Japan, Yokohama, Japan) were housed in pathogen-free rooms in a controlled environment under a 12 h light–dark cycle and allowed free access to laboratory chow and water. The experiments were approved by the Committee on Animal Research of Hiroshima University (approval no. A20-130) and conducted according to the Guide for the Care and Use of Laboratory Animals, 8th ed 2010 (National Institutes of Health, Bethesda, MD, USA).

### Cell line and culture condition

Enhanced green fluorescent protein-Lewis lung carcinoma (LLC) (Anti-Cancer Japan, Chiba, Japan) and KLN205 (Cell Resource Center for Biomedical Research Institute of Development, Aging, and Cancer, Tohoku University, Miyagi, Japan) cells were cultured in Dulbecco's modified Eagle medium (Thermo Fisher Scientific, Waltham, MA, USA) supplemented with 10% fetal bovine serum and 1% penicillin/streptomycin at 37 °C in a 5% CO_2_ incubator.

### Transfection and selection of stable green fluorescent protein (GFP)-expressing KLN205 cells

GFP and puromycin resistance genes were transfected into KLN205 cells using copGFP Control Lentiviral Particles (Santa Cruz Biotechnology, Dallas, TX, USA), following the manufacturer’s protocol. Cells were maintained in a puromycin-containing complete medium (Sigma-Aldrich, St. Louis, MO, USA).

### Mouse model of lung cancer with IP

Mice were anesthetized using mixed anesthetic agents, including medetomidine (0.3 mg/kg body weight; Kyoritsu Seiyaku, Tokyo, Japan), midazolam (4 mg/kg body weight; Sandoz K.K., Tokyo, Japan), and butorphanol (5 mg/kg body weight; Meiji Seika Pharma, Tokyo, Japan), and then administered bleomycin (2.0 mg/kg body weight; Nippon Kayaku, Tokyo, Japan) in saline through oropharyngeal aspiration (OA) using a micropipette [18]. Briefly, the mice were secured on a platform, their tongues were pulled out with forceps, and the bleomycin solution was placed onto the distal part of the oropharynx while the nasal cavity was closed gently with the technician’s fingers. 2 weeks later, the mice were transpleurally transplanted with lung cancer cells into their lungs [19]. Briefly, they were placed in the right lateral decubitus position, the hair of the thorax was trimmed, and a small skin incision was made on the left chest wall. Dissection was performed to expose the pleura and visualize lung movement. The tumor cell suspensions for injection into the lung were prepared with equal volumes of tumor cells (LLC 0.5 × 10^6^, KLN205 3.0 × 10^6^) in HBSS (Thermo Fisher Scientific) and Cultrex Reducer Growth Factor Basement Membrane Extract (R&D Systems, Minneapolis, MN, USA). A 29G 1 mL insulin syringe (Terumo, Tokyo, Japan) was used to inject the cells (50 μL) into the left lung. The needle was quickly advanced 7 mm into the lung and gently removed after the cell suspension injection. The skin was closed in a single layer using a 9 mm Autoclip (BD Biosciences, Franklin Lakes, NJ, USA). After tumor cell injection, mice were turned to the left lateral decubitus position.

### RNA extraction and sequencing

On day 21, tumor tissues obtained from the lung cancer alone (LC) and lung cancer with IP (IP + LC) models were homogenized using 1 mL of TRIzol reagent (Life Technologies, Grand Island, NY, USA). Total RNA was extracted using the RNeasy Mini kit (Qiagen, Hilden, Germany), following the manufacturer’s protocol. Bulk RNA barcoding and sequencing [20] were used for library preparation with some modifications: A barcoded oligo-dT-based primer [5′—GCCGGTAATACGACTCACTATAGGGAGTTCTACAGTCCGACGATCNNNNNNNNNNCCCCCCCCCTTTTTTTTTTTTTTTTTTTTTTTTV—3′; (10) N = UMI, (9) C = cell barcode] was used for single-stranded cDNA synthesis and Second Strand Synthesis Module (NEB, #E6111) was used for double-stranded cDNA synthesis. In-house MEDS-B Tn5 transposase [21, 22] was used for tagmentation, and libraries were amplified by 10 cycles of PCR using Phusion High-Fidelity DNA Polymerase (#M0530, Thermo Fisher Scientific) and primers (5'—AATGATACGGCGACCACCGAGATCTACACindexGTTCAGAGTTCTACAGTCCGA—3′, and 5′—CAAGCAGAAGACGGCATACGAGATindex GTCTCGTGGGCTCGGAGATGT—3′). Using NovaSeq6000 (Illumina, San Diego, CA, USA), 19 bp of the barcode reads (Read1) and 81 bp of insert reads (Read2) were obtained. Read1 was extracted using UMI-tools (ver.1.1.1, https://umi-tools.readthedocs.io/en/latest/) with the following command: “umi_tools extract -I read1.fastq –read2-in = read2.fastq –bc pattern = NNNNNNNNNNCCCCCCCCC –read2-stdout”. Adaptor and low-quality sequences were excluded, and read lengths below 20 bp were discarded using Trim Galore (ver. 0.6.7, https://www.bioinformatics.babraham.ac.uk/projects/trim_galore/) and mapped to GRCm38 using HISAT2 (ver. 2.2.1, http://daehwankimlab.github.io/hisat2/manual/). Read counts for each gene were obtained using featureCounts (ver. 2.0.1, https://www.rdocumentation.org/packages/Rsubread/versions/1.22.2/topics/featureCounts). UMI-tools removed UMI duplication with the following command: “umi_tools count –method = unique –per-gene –per-cell –gene-tag = XT.” Bioinformatics analyses were performed using Integrated Differential Expression and Pathway Analysis (ver. 0.96; http://bioinformatics.sdstate.edu/idep96/). Differentially expressed genes (DEGs) were extracted using an adjusted p-value < 0.1 as threshold values. Gene Ontology (GO) enrichment and Kyoto Encyclopedia of Genes and Genomes (KEGG) pathway analyses were performed to explore the biological functions of DEGs. In the GO enrichment analysis, the DEGs were assigned to one of three categories: biological processes, molecular functions, and cellular components. Raw RNA sequencing data were deposited in the NCBI for the Biotechnology Information Gene Expression Omnibus database (GEO GSE226270).

### Reverse transcription-quantitative PCR (RT-qPCR)

The extracted RNA was reverse-transcribed into cDNA using a High Capacity RNA-to-cDNA kit (Applied Biosystems, Foster City, CA, USA), following the manufacturer’s instructions. RT-qPCR was performed using Applied Biosystems 7500 Fast Real-Time PCR System (Applied Biosystems) to evaluate the expression of mouse *Hif1a* (Applied Biosystems; assay ID, Mm00468869_m1), *Timp1* (Applied Biosystems; assay ID, Mm01341361_m1), *Vegfa* (Applied Biosystems; assay ID, Mm_00437306_m1), and *Col1a1* (Applied Biosystems; assay ID, Mm00801666_g1), with the house-keeping gene *Actb* (Applied Biosystems; assay ID, Mm02619580_g1) used as a control.

### Hypoxia-inducible factor (HIF)-1α inhibition

Mice were injected with 50 mg/kg PX-478-2HCL (Selleck Chemicals, Houston, TX, USA) in double-distilled water intraperitoneally 2, 3, 4, 5, and 6 days after orthotopic implantation.

### HIF-1α activation

Mice were injected with 50 mg/kg dimethyloxalylglycine (DMOG) (Tokyo Chemical Industry, Tokyo, Japan) in double-distilled water intraperitoneally 2, 5, 8, and 11 days after orthotopic implantation.

### AsA treatment

Mice were injected with freshly prepared AsA in double-distilled water (4 g/kg; pH 7.0) intraperitoneally once daily for 2 days after orthotopic implantation.

### Tumor volume measurement

In the harvested lungs, tumor size was measured using calipers, and tumor volume (V) was calculated as follows: V = length × width × height × 0.5. The harvested mediastinal lymph nodes were weighed, and visible foci on the right lung surface were manually counted.

### Hydroxyproline assay

Lung hydroxyproline content was measured as described previously [23] to evaluate lung fibrosis.

### Complete blood count measurements

Complete blood cell counts were measured using whole blood collected in tubes with 50 mM ethylenediaminetetraacetic acid (Sigma-Aldrich, St. Louis, MO, USA) and a PCE-310 platform (Erma, Saitama, Japan).

### Histological analysis

Harvested murine lungs were fixed in 2% formalin (Nacalai Tesque, Kyoto, Japan) and embedded in paraffin. Sections (5 μm thick) were stained with hematoxylin–eosin or Masson’s trichrome.

### Immunohistochemical staining

Lung tissue sections were antigen-retrieved in 10 mmol/L sodium citrate (pH 6.0) using a microwave for 20 min, followed by staining with ENVISION + kit/horseradish peroxidase (HRP) (Dako, Tokyo, Japan) [24]. Rabbit anti-HIF-1α antibody (ab114977, 1:250; Abcam, Cambridge, UK), rabbit anti-tissue inhibitor of metalloproteinase-1 (TIMP-1) antibody (BS-0415R, 1:200, Bioss, Woburn, MA, USA), rabbit anti-VEGFA antibody (ab51745, 5 μg/mL; Abcam), or rabbit anti-Ki-67 antibody (no. 9027, 1:400; Cell Signaling, Beverly, MA, USA) was added after blocking endogenous peroxidase and proteins. The sections were then incubated with HRP-labeled anti-rabbit IgG antibody, followed by substrate chromogen. The sections were then counterstained with hematoxylin.

### Serum sampling

Blood samples were collected from the facial veins and centrifuged at 1000 ×*g* and 4 °C for 15 min to separate the cells. Serum was used to estimate cytokine concentrations.

### Enzyme-linked immunosorbent assay (ELISA)

HIF-1α, tissue inhibitor of metalloproteinase-1 (TIMP-1), and vascular endothelial growth factor A (VEGFA) levels (expressed as ng/mg protein) in the tumor tissue of the left lung homogenate were determined using the HIF-1α Sandwich ELISA kit (Cell Biolabs, San Diego, CA, USA), TIMP1-Cell Lysate Mouse ELISA kit (Thermo Fisher Scientific), and Mouse VEGF Quantikine ELISA kit (R&D Systems), respectively, following the manufacturer’s protocols. Total protein concentration in samples was measured using the Pierce™ bicinchoninic acid assay kit (Rockford, IL, USA). Serum transforming growth factor-β-1 (TGFβ-1) concentration (expressed as ng/mL) was measured using the TGFβ-1 ELISA kit (R&D Systems) following the manufacturer’s protocol.

### Flow cytometry and cell sorting

Mice were euthanized to obtain single-cell suspensions. Tumor tissues of the left lungs were excised, minced, and digested in RPMI1640 medium containing 1.0 mg/mL collagenase A (Roche Diagnostics, Basel, Switzerland) and 20 U/mL DNase I (Takara Bio Inc., Shiga, Japan) at 37 °C for 30 min. Red blood cells were lysed using the ammonium-chloride-potassium lysis buffer (Thermo Fisher Scientific). After blocking with anti-mouse CD16/32 antibodies (FcγR, clone 93, BioLegend, San Diego, CA, USA), cell suspensions were incubated with the appropriate dilutions of antibodies or their isotype-matched controls. Rat monoclonal antibodies for mouse CD3 (17A2), CD4 (GK1.5), CD8 (53–6.7), CD25 (PC61), CD31 (390), CD45 (30-F11), CD140a (APA5), CD206 (C068C2), and CD326 (G8.8) were purchased from BioLegend. The cells were fixed and permeabilized for intracellular staining using the Cytofix/Cytoperm kit (BD Biosciences, San Jose, CA, USA) before staining with αSMA (1A4, Thermo Fisher Scientific). Cells were analyzed and sorted using a BD FACS Aria II (BD Biosciences) or BD LSR Fortessa X-20 system (BD Biosciences). Data were analyzed using FlowJo (Tree Star, Inc., Ashland, OR, USA).

### Patients

This study was approved by the Ethics Committee of the Hiroshima University Hospital (E2022-0171). We retrospectively reviewed medical records of patients with non-small cell lung cancer (NSCLC) treated at the Hiroshima University Hospital. Patients who underwent surgery between January 2015 and December 2017 and those who received chemotherapy without an indication for surgery between January 2016 and March 2021 were enrolled. Information on patient characteristics before treatment, including chest computed tomography and pathology results, was obtained. An opt-out method was used to obtain patient consent.

### Statistical analyses

Statistical analyses were performed using JMP Pro 16 (SAS Institute, Cary, NC, USA). Mann–Whitney *U* test or Pearson’s chi-squared test was used for comparing two groups, and multiple Mann–Whitney *U* test with Bonferroni correction was used to compare three or more groups. Correlations between numerical variables were determined using Spearman’s correlations. Survival was evaluated using Kaplan–Meier approach. Statistical significance was set at *P* ≤ 0.05.

## Results

### Murine model of lung cancer with IP

**Fig. 1:**
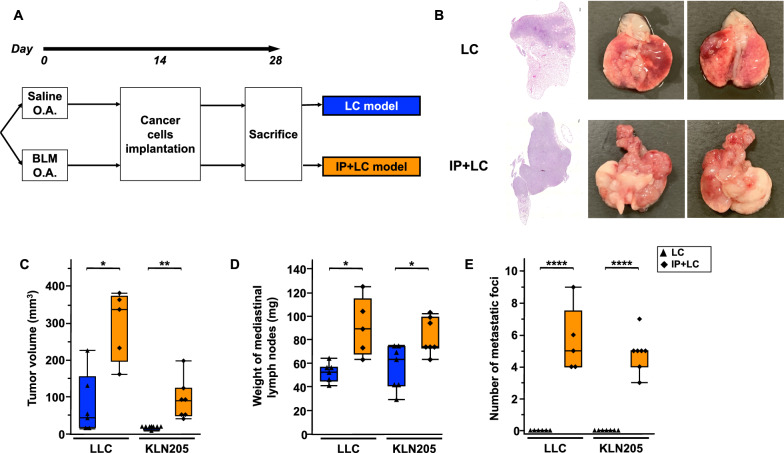
Novel murine model of lung cancer with interstitial pneumonia (IP) **A** Experimental scheme of the development of the mouse model of lung cancer with (IP + LC) or without (LC) interstitial pneumonia. LLC and KLN205 cells were the injected cancer cells. O.A., oropharyngeal administration. *BLM* bleomycin. **B** Hematoxylin and eosin staining of lung sections and macroscopic evaluation of the LC and IP + LC models on day 28. Scale bar: 300 μm. **C**–**E** Comparison of **C** tumor volume, **D** weight of mediastinal lymph nodes, and **E** number of metastatic foci between the LC and IP + LC models on day 28 (n = 5–7/group). *P < 0.05, **P < 0.01, ****P < 0.001

We developed a novel hybrid murine model of lung cancer with IP (IP + LC model) to validate the pathophysiological relationship between IP and lung cancer (Fig. 1A). Substantial tumor progression was observed macroscopically in the IP + LC model compared to that in the LC model (Fig. 1B). The tumor volume, mediastinal lymph node weight, and metastatic lung nodule count were significantly higher in the IP + LC model than in the LC model (Fig. 1C–E). No metastasis was observed in organs other than the lungs. These results were similar for LLC cell-transplanted C57BL/6 J and KLN205 cell-transplanted B6D2F1/Crl mice.

**Fig. 2:**
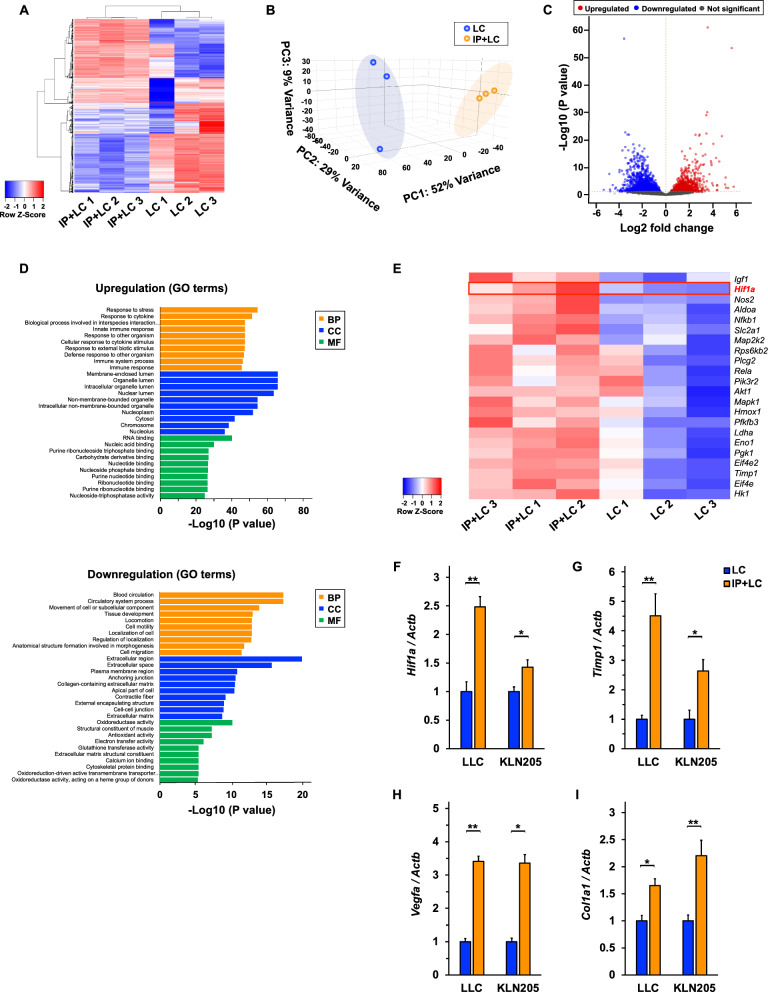
RNA sequencing and RT-qPCR of the LC and IP + LC models **A** Hierarchically clustered heatmap showing gene expression patterns. **B** Each dot represents one tumor sample subjected to principal component analysis (PCA). **C** Volcano plot of IP + LC and LC model gene expression profiles (n = 3/group). **D** Upregulated and downregulated terms in Gene Ontology (GO) enrichment pathway analysis when comparing the LC and IP + LC models. The terms are categorized into biological process (BP), cellular component (CC), and molecular function (MF). The y-axis shows the top 10 terms, and the x-axis shows the negative logarithm of the *P*-value. **E** Heatmap showing a portion of differentially expressed genes related to the HIF-1 signaling pathway between the models. **F**–**I** Comparison of mRNA expression of *Hif1a, Timp1*, *Vegfa,* and *Col1a1* in tumor tissues obtained from the LC and IP + LC models on day 21 (n = 4–5/group). Data are shown as mean ± SEM. *P < 0.05, **P < 0.01

In the RNA-sequencing analysis, 31,130 genes were quantitatively detected. Hierarchical clustering and principal component analysis showed that the IP + LC and LC groups were clearly distinct, indicating a difference in gene expression between the groups (Fig. 2A, B). In total, 3215 DEGs (1478 significantly upregulated and 1737 significantly downregulated) were identified between these groups (Fig. 2C). GO analysis revealed that DEGs associated with the immune system were upregulated, and those associated with intercellular adhesion were downregulated (Fig. 2D). KEGG pathway analysis revealed the HIF-1 signaling pathway as a pathway associated with both lung cancer and IP (Fig. 2E). *Hif1a* mRNA expression was compared between the healthy control, IP model (Additional file 1: Fig. S1A), LC model, and IP + LC model. Both the IP and LC models showed significantly higher *Hif1a* expression when compared to the healthy control. Moreover, the IP + LC model showed higher *Hif1a* expression than both the IP and LC models (Additional file 1: Fig. S1B). In addition, RT–qPCR was performed to compare *Hif1a* expression between the sorted cancer cells (GFP + /CD45-) from the IP + LC and LC models. Cells from the IP + LC model showed significantly higher *Hif1a* expression than the LC model (Additional file 1: Fig. S2).

In the HIF-1 pathway, we focused on two mRNAs: *Timp1*, which is involved in the extracellular matrix, barrier function, and cell proliferation, and *Vegfa*, which is involved in angiogenesis and fibrosis. In addition, we focused on *Col1a1* as the factor associated with fibrosis. RT-qPCR was performed to validate the RNA sequencing results. The IP + LC group showed significantly higher *Hif1a*, *Timp1*, *Vegfa*, and *Col1a1* expression than the LC group (Fig. 2F–I). In addition, both the IP and LC models showed significantly higher *Timp1*, *Vegfa*, and *Col1a1* expression than the healthy control. Moreover, the IP + LC model showed higher expression of these mRNAs than both the IP and LC models (Additional file 1: Fig. S1C–E).

### Inhibition of the HIF-1 pathway as a therapeutic strategy for lung cancer with IP

**Fig. 3:**
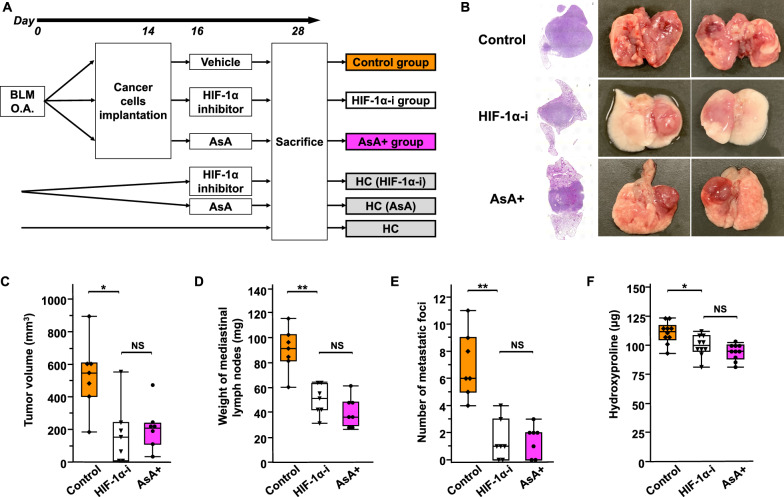
HIF-1α pathway inhibitor as a therapeutic strategy for lung cancer with interstitial pneumonia (IP) **A** Experimental scheme of comparison of treatments in the IP + LC model. The injected cancer cells were LLC cells. O.A., oropharyngeal administration. *BLM* bleomycin, AsA ascorbic acid, *HIF-1α* hypoxia-inducible factor 1α. *HIF-1α-i*, HIF-1α inhibitor. *HC* Healthy control. **B** Hematoxylin and eosin staining of lung sections and macroscopic evaluation obtained from the Control, HIF-1α-i, and AsA + groups on day 28. Scale bar: 300 μm. **C**–**E** Comparison of **C** tumor volume, **D** weight of mediastinal lymph nodes, and **E** number of metastatic foci among the Control, HIF-1α-i, and AsA + groups on day 28 (n = 7/group). NS represents not significant. *P < 0.05, **P < 0.01. **F** Comparison of hydroxyproline levels among the Control, HIF-1α-i, and AsA + groups on day 28 (n = 10/group). NS represents not significant. *P < 0.05

We hypothesized that HIF-1 inhibitors may be a potential treatment for lung cancer with interstitial pneumonia, and AsA, which promotes HIF-1α degradation, may be an alternative therapeutic candidate. Therefore, we compared the efficacy of the HIF-1α inhibitor (HIF-1α-i) PX-478-2HCL to that of AsA in the IP + LC model (Fig. 3A). The AsA dose was the same as that in previous studies [25, 26]. Compared to those in the control group, the enhanced tumor progression was suppressed (Fig. 3B) and the volume of primary tumors was significantly decreased to the same degree in the HIF-1α-i and AsA ( +) groups (Fig. 3C). Similar results were observed for the mediastinal lymph node weight, metastatic lung nodule count, and hydroxyproline levels (Fig. 3D–F). Overall, the antitumor and antifibrotic effects of HIF-1α-i and AsA on the IP + LC model were comparable.

Body weights (BW) and complete blood counts (CBC) were measured to investigate the side effects of the HIF-1α inhibitor and AsA. The HIF-1α-i, but not the AsA + , group had significantly lower BW than the control group (Additional file 1: Fig. S3A). The HC (HIF-1α-i), but not the HC (AsA), group had significantly lower red blood cell counts and hemoglobin levels than the HC group (Additional file 1: Fig. S3B–C). Similarly, the HIF-1α-i, but not the AsA + , group also had significantly lower red blood cell counts and hemoglobin levels than the control group (Additional file 1: Fig. S3B–C). In addition, the HC (HIF-1α-i), but not the HC (AsA), group had significantly lower white blood cell counts than the HC group (Additional file 1: Fig. S3D). No significant differences were observed in the platelet counts between groups (Additional file 1: Fig. S3E).

### Effect of AsA on lung cancer with IP

**Fig. 4:**
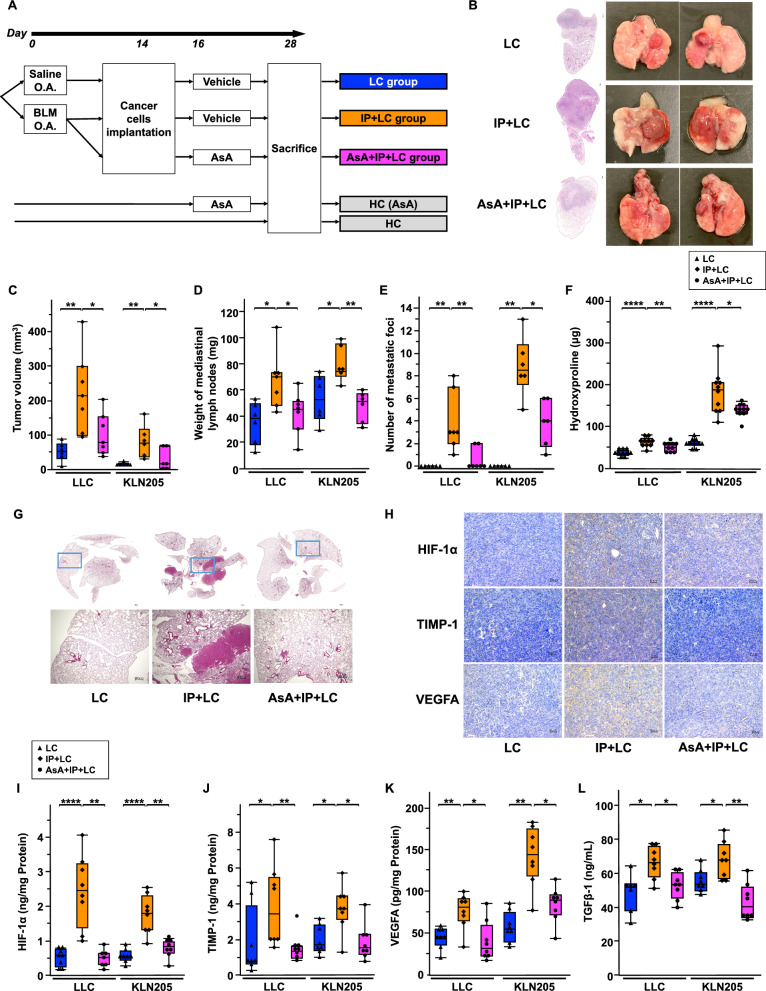
Effect of AsA on lung cancer with interstitial pneumonia (IP). **A** Experimental scheme comparing tumor progression and lung fibrosis among the LC, IP + LC, and AsA + IP + LC groups. The injected cancer cells were LLC and KLN205 cells. O.A., oropharyngeal administration. BLM, bleomycin. AsA, ascorbic acid. HC, Healthy control. **B** Hematoxylin and eosin staining of lung sections and macroscopic evaluation of the LC, IP + LC, and AsA + IP + LC groups on day 28. The results are shown for LLC. Scale bar: 300 μm. **C**–**E** Comparison of **C** tumor volume, **D** weight of mediastinal lymph nodes, and **E** number of metastatic foci among the LC, IP + LC, and AsA + IP + LC groups on day 28 (n = 6–7/group). *P < 0.05, **P < 0.01. **F** Comparison of hydroxyproline levels among the LC, IP + LC, and AsA + IP + LC groups on day 28 (n = 10/group). *P < 0.05, **P < 0.01, ****P < 0.001. **G** Masson’s trichrome staining of lung tissue sections obtained from the LC, IP + LC, and AsA + IP + LC groups on day 28. Scale bar: 300 μm. **H** Immunohistochemical staining of LC, IP + LC, and AsA + IP + LC group tumor tissues on day 28, with anti-HIF-1α, anti-TIMP-1, and anti-VEGFA antibodies. The results are shown for LLC cells. AsA, ascorbic acid. HIF-1α, hypoxia-inducible factor 1α. TIMP-1, tissue inhibitor of metalloproteinase-1. VEGFA, vascular endothelial growth factor A. Scale bar: 50 μm. **I**–**K** Comparison of the concentration of **I** HIF-1α, **J** TIMP-1, and (K) VEGFA protein in the tumor tissue among the LC, IP + LC, and AsA + IP + LC groups on day 28 (n = 7–8/group). *P < 0.05, **P < 0.01, ****P < 0.001. **L** Comparison of serum TGFβ-1 protein concentration among the LC, IP + LC, and AsA + IP + LC groups on day 28 (n = 8/group). *P < 0.05, **P < 0.01

First, we analyzed the effect of AsA in lung cancer alone (Additional file 1: Fig. S4A) and IP alone (Additional file 1: Fig. S5A) models and found that AsA suppressed cancer progression (Additional file 1: Fig. S4B–D) and pulmonary fibrosis (Additional file 1: Fig. S5B, C) in both models. Next, we assessed the effect of AsA in the novel IP + LC model using two different cancer cell lines (LLC and KLN205) (Fig. 4A). The enhanced tumor progression was inhibited in the AsA + IP + LC group compared to that in the IP + LC group (Fig. 4B). Primary tumor volume was significantly higher in the IP + LC group than in the LC group, whereas it was significantly decreased in the AsA + IP + LC group (Fig. 4C). Similar results were observed for the mediastinal lymph node weight and the metastatic lung nodule count, regardless of the murine species or lung cancer histological type (Fig. 4D, E). Moreover, hydroxyproline levels were higher in the IP + LC group than in the LC group and were significantly lower in the AsA + IP + LC group than in the IP + LC group (Fig. 4F). Histological examination of the right lung revealed overlapping pulmonary fibrosis and intrapulmonary metastases in the IP + LC group but not in the LC group (Fig. 4G). Moreover, the AsA + IP + LC group showed significantly reduced metastases and fibrosis compared to the IP + LC group. The IP + LC group had significantly higher red blood cell counts, but not hemoglobin levels than the LC group (Additional file 1: Fig. S6B–C). No significant differences were observed in the other parameters between groups (Additional file 1: Fig. S6A–E). Cytokine analysis was further performed to examine the effects of AsA. Immunohistochemical staining revealed increased expression of HIF-1α, TIMP-1, and VEGFA in the IP + LC group compared to that in the LC group; however, this increased expression was suppressed by AsA (Fig. 4H, Additional file 1: Fig. S7). Histological findings were confirmed at the protein level using ELISA; HIF-1α, TIMP-1, and VEGFA levels in the tumor tissue were significantly higher in the IP + LC group than in the LC group (Fig. 4I–K), and these were reduced by treatment with AsA, regardless of the murine species or lung cancer histological type. Similar results were observed for the levels of serum TGFβ-1, which is involved in fibrosis and tumor progression (Fig. 4L).

### IP-mediated changes in the tumor microenvironment

**Fig. 5:**
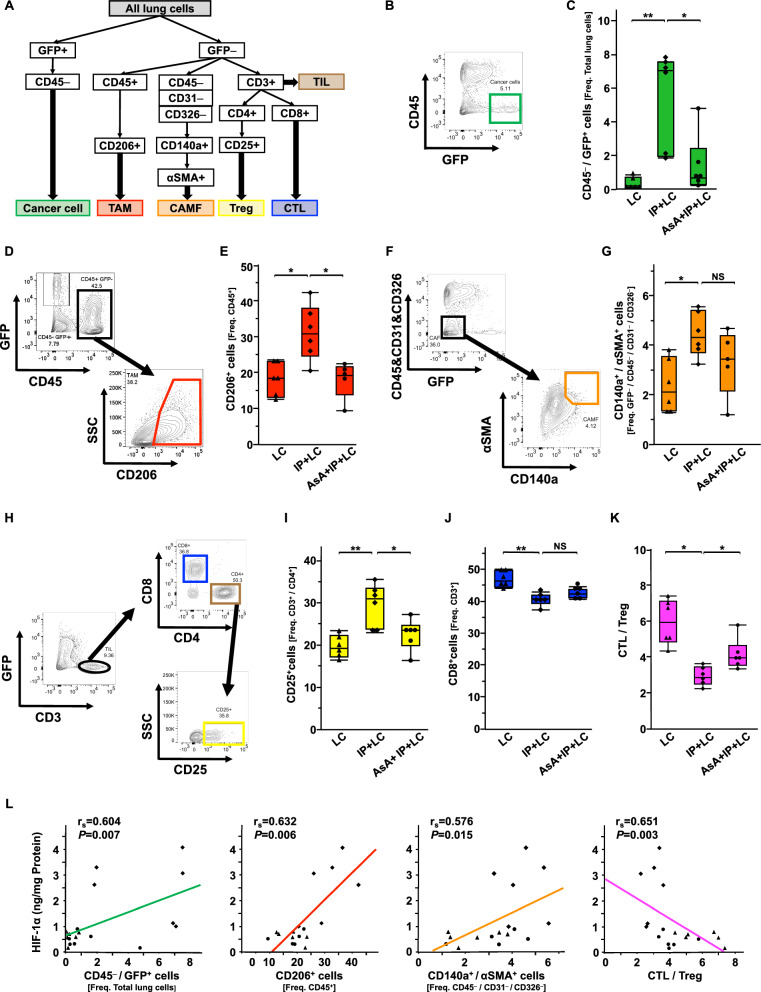
Interstitial pneumonia (IP)-mediated changes in the tumor microenvironment **A** Flow cytometric strategy to define cancer cells, tumor-associated macrophages *TAM *cancer-associated myofibroblasts, *CAMF* tumor-infiltrating lymphocytes ,*TIL* regulatory T cells (Treg), and cytotoxic T lymphocytes (CTL). The injected cancer cells were LLC cells. **B** Representative gating image of cancer cells (GFP + /CD45-) in all lung cells. **C** Comparison of cancer cell fraction among the LC, IP + LC, and AsA + IP + LC groups on day 28 (n = 6/group). *P < 0.05, **P < 0.01. AsA, ascorbic acid. **D** Representative gating image of TAM (GFP-/CD45 + /CD206 +) in all lung cells. **E** Comparison of TAM fraction among the LC, IP + LC, and AsA + IP + LC groups on day 28 (n = 5–6/group). *P < 0.05. **F** Representative gating image of CAMF (CD140a + /αSMA +) in lung GFP-/CD45-/CD31-/CD326- cells. **G** Comparison of CAMF fraction among the LC, IP + LC, and AsA + IP + LC groups on day 28 (n = 5–6/group). NS represents not significant. *P < 0.05. **H** Representative gating images of CTL (CD8 +) in TIL (GFP-/CD3 +) and Tregs (CD25 +) in CD4 + TIL. **I**–**K** Comparison of **I** Treg fraction, **J** CTL fraction, and **K** CTL/Treg ratio among the LC, IP + LC, and AsA + IP + LC groups on day 28 (n = 6/group). NS represents not significant. *P < 0.05. **P < 0.01. **L** Correlations between HIF-1α expression in the tumor tissue and cancer cell fraction, TAM fraction, CAMF fraction, and CTL/Treg ratio on day 28. HIF-1α, hypoxia-inducible factor 1α

Flow cytometry was performed using lung tumor tissues to examine how IP alters the tumor microenvironment. The flow cytometric definitions of each cell fraction were as follows: lung cancer cell (GFP + /CD45−), tumor-associated macrophages (TAM; GFP-/CD45 + /CD206 +), cancer-associated myofibroblasts (CAMF; GFP-/CD45-/CD31-/CD326-/CD140a + /αSMA +), regulatory T cells (Treg; GFP−/CD3 + /CD4 + /CD25 +), and cytotoxic T lymphocytes (CTL; GFP-/CD3 + /CD8 +) (Fig. 5A). The cancer cell fraction was significantly higher in the IP + LC group than in the LC group, and AsA significantly decreased it (Fig. 5B, C). Similar results were observed for the TAM fraction (Fig. 5D, E). The CAMF fraction was significantly higher in the IP + LC group than in the LC group. However, the difference between the IP + LC and AsA + IP + LC groups was not significant (Fig. 5F, G). The Treg fraction was also significantly higher in the IP + LC group than in the LC group, but it was decreased by AsA (Fig. 5H, I). In contrast, the CTL fraction was significantly lower in the IP + LC group than in the LC group; however, the difference between the IP + LC and AsA + IP + LC groups was not significant (Fig. 5H, J). The CTL/Treg ratio was significantly lower in the IP + LC group than in the LC group, which was reversed by AsA (Fig. 5K). In addition, the relationship between the HIF-1 signaling pathway and the tumor microenvironment was examined. Significant correlations were observed between HIF-1α and the cancer cell fraction, TAM fraction, CAMF fraction, and CTL/Treg ratio (Fig. 5L). These results were similar for LLC cell-transplanted C57BL/6 J and KLN205-cell transplanted B6D2F1/Crl mice (Additional file 1: Fig. S8A–G).

### HIF-1α plays a key role in AsA treatment

**Fig. 6:**
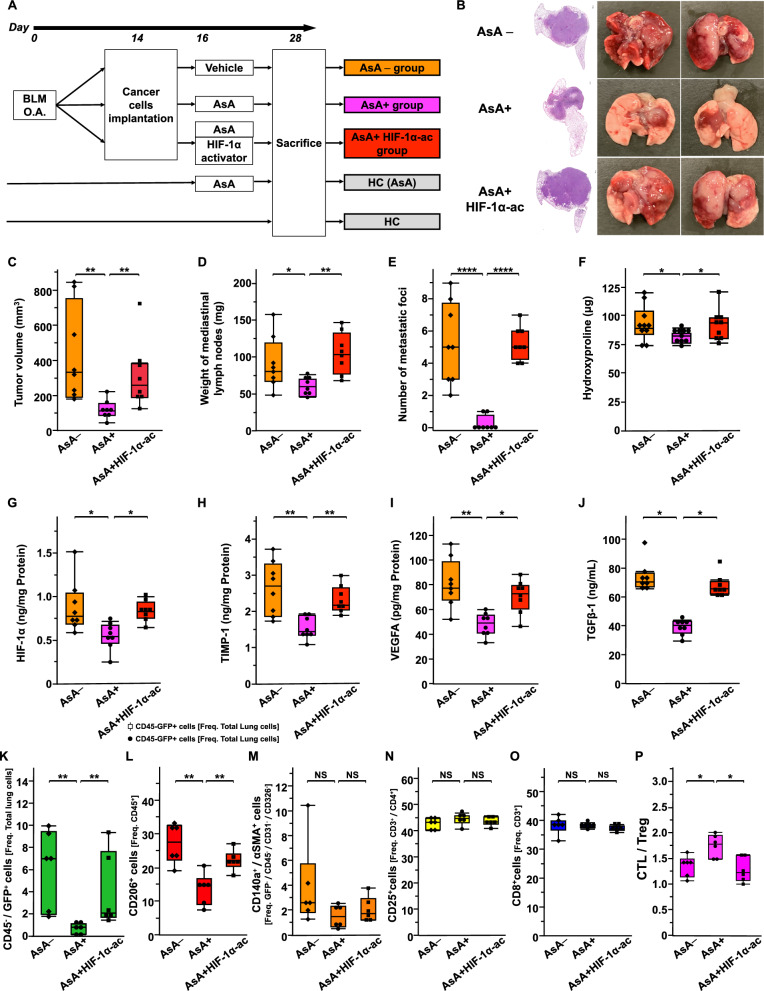
HIF-1α plays a key role in treatment with AsA **A** Experimental scheme comparing tumor progression and lung fibrosis among the AsA–, AsA + , and AsA + HIF-1α-ac groups. The injected cancer cells were LLC cells. *OA*, oropharyngeal administration, *BLM* bleomycin, *AsA* ascorbic acid, *HIF-1α* hypoxia-inducible factor 1α, *HIF-1α-ac* HIF-1α activator, *HC* Healthy control. **B** Hematoxylin and eosin staining of lung sections and macroscopic evaluation results from the AsA−, AsA + , and AsA + HIF-1α-ac groups on day 28. Scale bar: 300 μm. **C**–**E** Comparison of **C** tumor volume, **D** weight of mediastinal lymph nodes, and **E** number of metastatic foci among the AsA−, AsA + , and AsA + HIF-1α-ac groups on day 28 (n = 8/group). *P < 0.05, **P < 0.01, ****P < 0.001. **F** Comparison of hydroxyproline level among the AsA−, AsA + , and AsA + HIF-1α-ac groups on day 28 (n = 10/group). *P < 0.05. **G**–**I** Comparison of the concentrations of **G** HIF-1α, **H** TIMP-1, and (I) VEGFA proteins in the tumor tissue among the AsA−, AsA + , and AsA + HIF-1α-ac groups on day 28 (n = 8/group). TIMP-1, tissue inhibitor of metalloproteinase-1. VEGFA, vascular endothelial growth factor A. *P < 0.05, **P < 0.01. **J** Comparison of the concentrations of serum TGFβ-1 protein among the AsA−, AsA + , and AsA + HIF-1α-ac groups on day 28 (n = 8/group). *P < 0.05. **K**–**P** Comparison of **K** cancer cell fraction, **L** tumor-associated macrophages (TAM) fraction, **M** cancer-associated myofibroblasts (CAMF) fraction, **N** regulatory T cells (Treg) fraction, **O** cytotoxic T lymphocytes (CTL) fraction, and **P** CTL/Treg ratio among the AsA−, AsA + , and AsA + HIF-1α-ac groups on day 28 (n = 6/group). *NS* represents not significant. *P < 0.05, **P < 0.01

To confirm that HIF-1α degradation is a key mechanism underlying the efficacy of AsA treatment in the IP + LC model, IP + LC models treated with or without AsA (AsA [ +] and AsA [−] groups) were compared with the IP + LC model treated with both AsA and the HIF-1α activator DMOG (AsA + HIF-1α-ac group) (Fig. 6A). DMOG diminished the suppressed tumor progression macroscopically observed in the AsA ( +) group (Fig. 6B). Consistently, the reduced tumor volume, mediastinal lymph node weight, metastatic lung nodule count, and hydroxyproline levels observed in the AsA ( +) group were also diminished by DMOG (Fig. 6C–F). There were no significant changes in BW or CBC between groups (Additional file 1: Fig. S9A–E).

The HIF-1α level in the tumor tissue was significantly lower in the AsA ( +) group than in the AsA (−) group but higher in the AsA + HIF-1α-ac group than in the AsA ( +) group, suggesting that DMOG reversed the decrease in HIF-1α level in the IP + LC model administered AsA (Fig. 6G). Similar results were obtained for TIMP-1, VEGFA, and serum TGFβ-1 levels (Fig. 6H–J). The effects of DMOG on the tumor microenvironment were also examined using flow cytometry (Fig. 6K–P). DMOG diminished the suppressed cancer cell and TAM fractions in the AsA ( +) group. The CAMF fraction was not significantly lower in the AsA ( +) group than in the AsA (−) group, and the difference between the AsA ( +) and AsA + HIF-1α-ac groups was also not significant. However, a significant change was observed in the CTL/Treg ratio.

### Prognosis of patients with NSCLC with and without IP

**Fig. 7:**
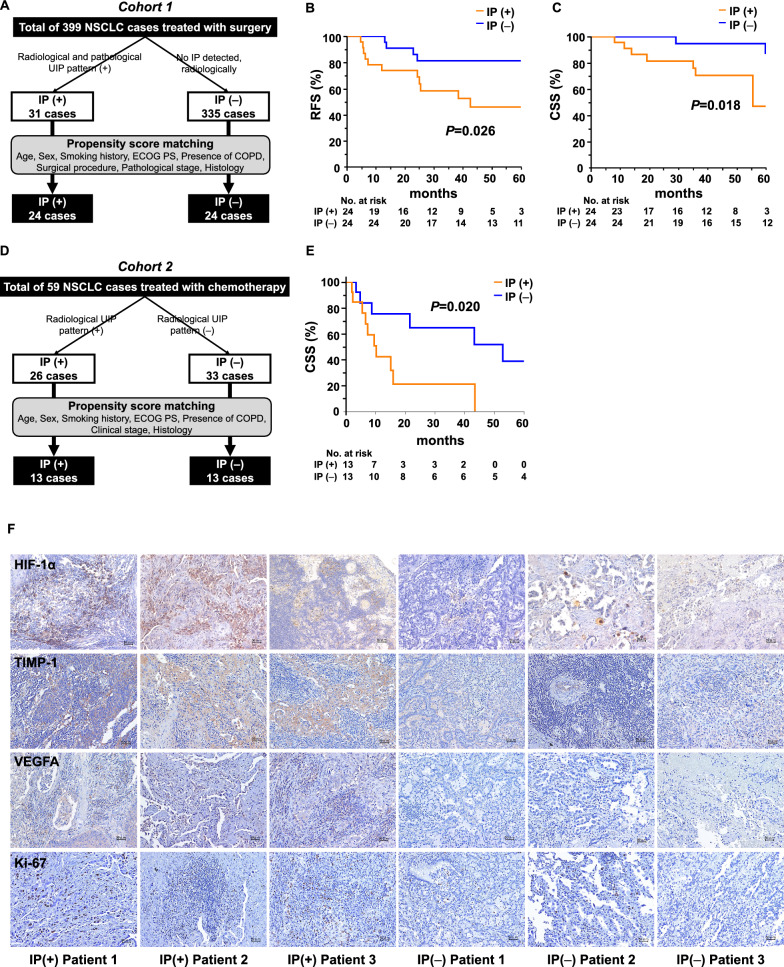
Prognosis of patients with non-small cell lung cancer with and without interstitial pneumonia (IP) **A** Definition of patients with lung cancer with IP [IP ( +)] and without IP [IP (−)] among the enrolled patients with NSCLC treated with surgery (cohort 1). NSCLC, non-small cell lung cancer. UIP, usual interstitial pneumonia. ECOG PS, Eastern Cooperative Oncology Group Performance Status. COPD, chronic obstructive pulmonary disease. **B**–**C** Kaplan–Meier survival curves for **B** recurrence-free survival (RFS) and **C** cause-specific survival (CSS) periods between the IP ( +) and IP (−) groups in cohort 1. **D** Definition of patients with lung cancer with IP [IP ( +)] and without IP [IP (−)] among the enrolled patients with NSCLC treated with chemotherapy (cohort 2). **E **Kaplan–Meier survival curves for CSS between the IP ( +) and IP (−) groups in cohort 2. **F** HIF-1α, TIMP-1, VEGFA, and Ki-67 expression in tumor specimens of the IP ( +) and IP (−) groups in cohort 2. Representative images of tumor specimens stained with an antibody against human HIF-1α, TIMP-1, VEGFA, and Ki-67 via immunohistochemistry

To validate the results obtained from animal experiments, we retrospectively studied two cohorts of patients with NSCLC: patients with early-stage NSCLC who underwent surgery (cohort 1) and patients with advanced NSCLC who received chemotherapy alone (excluding immune checkpoint and tyrosine kinase inhibitors) without an indication for surgery (cohort 2). Of the 399 patients in cohort 1 (Fig. 7A), 31 with radiologically confirmed IP and a pathological usual interstitial pneumonia pattern were included in the IP ( +) group, and 335 patients lacking radiological evidence of IP were included in the IP (−) group. Thirty-three patients with radiologically confirmed IP but not the pathological usual interstitial pneumonia patterns were excluded. Propensity score matching was performed for age, sex, smoking history, Eastern Cooperative Oncology Group Performance Status, presence of chronic obstructive pulmonary disease, surgical procedure, pathological staging, and histological type. Finally, 24 IP ( +) and 24 IP () cases were analyzed.

After matching, the Krebs von den Lungen-6 and carcinoembryonic antigen levels were significantly higher in the IP ( +) group than in the IP (−) group (Table 1). The postoperative recurrence-free survival,cause-specific survival (CSS), and overall survival (OS) periods were significantly shorter in the IP ( +) group when compared with the IP (−) group (Fig. 7B, C and Additional file 1: Fig. S10A). HIF-1α and Ki-67 expression in the resected lung tissue were representatively increased in the cancer cells from patients with underlying IP (Additional file 1: Fig. S10B). The IP ( +) group showed significantly higher Ki-67 expression in the tumor area of the resected lung than the IP (−) group (Additional file 1: Fig. S10C, *P* = 0.040, 47.0 ± 20.0 vs. 31.2 ± 25.2).

**Table 1:** Characteristics of patients who underwent surgery

Patient characteristic	IP ( +)	IP (−)	*P*
Participants, n	n = 24	n = 24	
Age (years)	74.0 (69.5–77.0)	72.5 (67.2–78.0)	0.901
Sex, male/female	20/4	23/1	0.143
ECOG PS, 0/1	18/6	18/6	1.000
Smoking history, pack-years	48.0 (26.2–75.0)	60.0 (42.5–87.5)	0.301
COPD, ±	7/17	9/15	0.539
Pathological stage, I/II/IIIA/IIIB	16/3/4/1	18/4/2/0	0.507
Histology			0.933
Adenocarcinoma	8	8	
Squamous cell carcinoma	11	10	
Others	5	6	
Surgical procedure			0.944
Lobectomy	10	11	
Segmentectomy	6	6	
Partial resection	8	7	
KL-6, U/mL	482.0 (306.7–771.5)	262.5 (204.7–341.0)	< 0.001
CEA	4.9 (2.8–9.8)	3.2 (2.4–3.9)	0.012
CYFRA	4.0 (2.3–4.6)	2.5 (1.8–4.9)	0.132

Data are expressed as frequency or median (interquartile range). *IP* interstitial pneumonia, *ECOG PS* eastern cooperative oncology group performance status, *COPD* chronic obstructive pulmonary disease, *KL-6* Krebs von den Lungen-6, *CEA* carcinoembryonic antigen, *CYFRA* cytokeratin 19 fragment

**Fig. 8:**
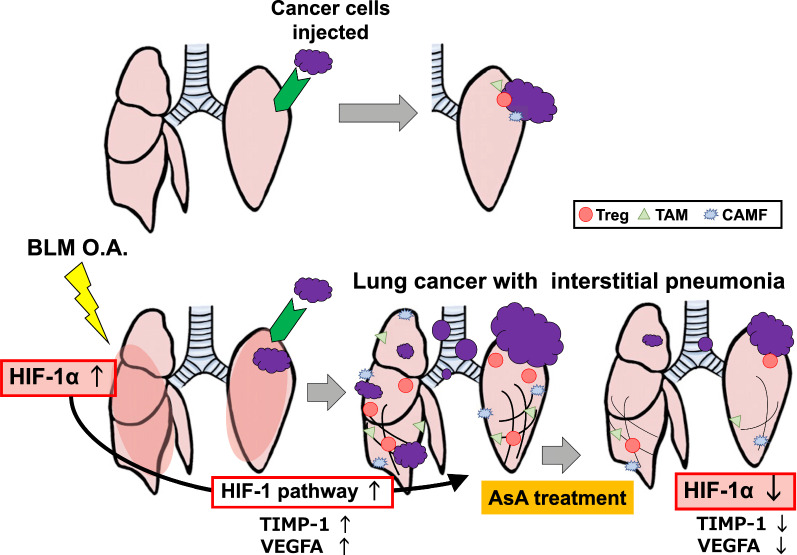
Summary of the study *BLM* bleomycin. *OA* oropharyngeal administration, *AsA* ascorbic acid, *Treg* regulatory T cells. *TAM* tumor-associated macrophages, *CAMF* cancer-associated myofibroblasts, *HIF-1α* hypoxia-inducible factor 1α, *TIMP-1* tissue inhibitor of metalloproteinase-1, *VEGFA* vascular endothelial growth factor A

In cohort 2, the enrolled 59 patients were radiologically divided into 26 with IP (IP [ +] group) and 33 without IP (IP [−] group) (Fig. 7D). Propensity score matching was performed for age, sex, smoking history, Eastern Cooperative Oncology Group Performance Status, presence of chronic obstructive pulmonary disease, clinical staging, and histological type. Finally, 13 IP ( +) and 13 IP (−) cases were analyzed. After matching, the Krebs von den Lungen-6 and cytokeratin 19 fragment levels were significantly higher in the IP ( +) group than in the IP (−) group (Table 2). Similar to that seen in cohort 1, the CSS and OS were significantly shorter in the IP ( +) group than in the IP (−) group (Fig. 7E and Additional file 1: Fig. S10D). In cohort 2, HIF-1α, TIMP-1, VEGFA, and Ki-67 expression of surgical lung biopsy cases was representatively increased in the cancer cells underlying IP (Fig. 7F). The IP-mediated lung cancer progression observed in the animal model and human cohort is summarized in Fig. 8.

**Table 2:** Characteristics of patients who underwent chemotherapy

Patient characteristic	IP ( +)	IP (−)	*P*
Participants, n	n = 13	n = 13	
Age (years)	69.0 (68.0–74.0)	68.0 (65.5–72.5)	0.254
Sex, male/female	12/1	11/2	0.535
ECOG PS			
0/1/2	7/4/2	7/5/1	0.798
Smoking history			
Pack-years	50.0 (45.0–63.0)	47.0 (42.5–73.5)	0.938
COPD			
+/−	12/1	11/2	0.535
Clinical stage			
IIIA/IIIB/IVA/IVB	1/0/7/5	1/0/10/2	0.394
Histology			0.489
Adenocarcinoma	11	12	
Squamous cell carcinoma	1	1	
Others	1	0	
KL-6, U/mL	949.0 (446.0–1683.0)	461.0 (214.0–771.0)	0.039
CEA	10.5 (5.5–21.6)	42.7 (6.1–307.4)	0.127
CYFRA	9.7 (3.5–14.3)	5.2 (1.7–5.8)	0.036

Data are expressed as frequency or median (interquartile range). *IP* interstitial pneumonia, *ECOG PS* eastern cooperative oncology group performance status, *COPD* chronic obstructive pulmonary disease, *KL-6* Krebs von den Lungen-6, *CEA* carcinoembryonic antigen, *CYFRA* cytokeratin 19 fragment

## Discussion

In the present study, we developed a novel murine model to investigate the effect of IP on lung cancer and the tumor microenvironment. In addition to rapid primary tumor growth and increased mediastinal lymph node weight, we observed lung metastases in the IP + LC mouse model but not in the LC model. These results are consistent with previous reports of rapid tumor growth in lung cancer with IP [27] and our findings of early postoperative recurrence and short overall survival in patients with lung cancer accompanied by IP. Moreover, the IP ( +) and IP (−) groups showed a significant difference in the Ki-67 score of the tumor cells, suggesting that chronic microenvironmental changes in the lungs due to IP can increase the malignant potential of lung cancer. In the IP + LC model, metastatic areas generally overlapped the lung fibrosis areas induced by bleomycin, which is consistent with the fact that, in chest computed tomography scans of lung cancer with IP, most tumors are observed in fibrotic areas [8]. Therefore, the IP + LC model developed in this study mimics human pathophysiology. Furthermore, in the IP + LC model, metastasis was observed only in the lungs, suggesting a close relationship between lung inflammation or fibrosis and lung cancer.

In the present study, RNA sequencing revealed an altered immune system, downregulated intercellular adhesion, and enhanced HIF-1 signaling pathway in the IP + LC model compared to the LC model. HIF-1 is a hypoxia-dependent inducer of erythropoietin in the hepatocarcinoma cell line Hep3B [28]. In many solid tumors, HIF-1 activation allows cancer cells to adapt to their environment via metabolic reprogramming and angiogenesis and increases the metastatic potential and resistance to chemotherapy and radiotherapy via epithelial–mesenchymal transition [29–32]. Furthermore, in patients with NSCLC who underwent surgery, the group with a high HIF-1α score in the resected lungs exhibited a poorer prognosis than the low HIF-1α score group [33]. Activated HIF-1α participates in murine bleomycin-induced pulmonary fibrosis [34] and human idiopathic pulmonary fibrosis [35]. As expected, the elevated *Hif1α* expression in the IP and LC models (compared to healthy mice) was also observed. Furthermore, we found that *Hif1a* expression was further elevated in the IP + LC model than that seen in IP or LC alone. Thus, the HIF-1 signaling pathway was shown to be associated with both lung cancer and IP. Therefore, we focused on HIF-1α as a promising key player in IP-mediated lung cancer progression and tumor microenvironmental changes, and demonstrated that a HIF-1α inhibitor was effective against lung cancer with IP. Administering pharmacological concentrations of AsA (an alternative to HIF-1 inhibitors) inhibited the progression of both cancer and underlying pulmonary fibrosis, with effects comparable to those seen with HIF-1α inhibitors. In addition, AsA administration did not appear to cause any side effects such as BW or CBC changes. AsA level is tightly regulated in the bloodstream by renal excretion [36]. AsA regulates the transcriptional activity of HIF-1 and HIF-2 in cells; it promotes HIF-1 degradation by enhancing iron-dependent dioxygenase activation, which hydroxylates critical proline and asparagine residues in HIF-1α [37]. Although physiological AsA concentrations may not regulate HIF-1 in cancer cells, pharmacological AsA concentrations reportedly suppress HIF-1 [38, 39]. Notably, the anticancer effects of AsA are not limited to HIF-1α suppression; AsA contributes to glycolysis attenuation in cancer cells [25], causing DNA damage via reactive oxygen species [26] due to redox imbalance. Our experiments using DMOG, which inhibits the effects of AsA, demonstrated that the anticancer effects of AsA mainly depend on HIF-1α suppression. However, the effects of AsA on IP are poorly understood. Although clinical studies are limited [40], animal studies have reported the usefulness of AsA in a paraquat-induced pulmonary fibrosis model [41] and decreased AsA concentration in the lungs of bleomycin-induced pulmonary fibrosis mice [42]. Therefore, pharmacological AsA concentrations might inhibit HIF-1α and suppress cancer cell proliferation while preventing IP progression.

In the present study, we demonstrated that HIF-1α upregulation in the IP + LC model was suppressed by AsA. In association with tumor and microenvironmental cells, HIF-1 induces angiogenesis via HIF-1-dependent expression of VEGFA and platelet-derived growth factor B, aids cancer cell survival by mediating extracellular matrix remodeling and metabolic reprogramming in cancer-associated fibroblasts, and suppresses adaptive immune system via Treg and TAM recruitment and activation [43]. The contribution of the interaction between tumors and microenvironmental cells to cancer progression is widely accepted; however, few studies have examined the tumor microenvironment in lung cancer with IP. In the present study, we confirmed that IP affects the tumor microenvironment, and this altered tumor microenvironment is significantly correlated with HIF-1α expression. Furthermore, AsA corrected the imbalance in the tumor microenvironment.

This study had some limitations. First, statistical analyses of HIF-1α expression and the tumor microenvironment in human lung cancer with IP were not performed. Most advanced lung cancers are diagnosed using small tissue samples obtained via transbronchial lung biopsy; therefore, we could not obtain sufficient tissue samples for this analysis. Second, our human cohort was retrospective, with a small sample size. Hence, prospectively examining HIF-1α expression in more cases of human lung cancer with IP is required. Finally, the AsA dose in the IP + LC model was relatively high, and the method of AsA administration needs to be considered for human adaptation.

## Conclusions

In the present study, using a novel disease model of lung cancer with IP and a human cohort, we demonstrated that the HIF-1 pathway plays a critical role in IP-mediated lung cancer progression and can thus serve as a therapeutic target. Overall, the study provides important insights into improving the management of patients with lung cancer and IP.

### Supplementary Information

**Additional file 1: Fig. S1.** Comparison of gene expression in each model. **Fig. S2.** Comparison of *Hif1a* expression between the sorted cancer cells from the IP+LC and LC models. **Fig. S3.** Comparison of side effects in each group of Fig. 3. **Fig. S4.** Effect of AsA on lung cancer alone model. **Fig. S5.** Effect of AsA on IP alone model. **Fig. S6.** Comparison of side effects in each group of Fig. 4. **Fig. S7.** Effect of AsA on lung cancer with interstitial pneumonia (IP). **Fig. S8.** Interstitial pneumonia (IP)-mediated changes in the tumor microenvironment based on flow cytometry. **Fig. S9.** Comparison of side effects in each group of Fig. 6. **Fig. S10.** Prognosis of patients with non-small cell lung cancer with and without interstitial pneumonia (IP).

## Data Availability

The data generated in this study and materials are available from the corresponding author upon reasonable request. Raw RNA sequencing data are deposited in the NCBI for the Biotechnology Information Gene Expression Omnibus database (GEO GSE226270).

## Abbreviations

AsA: Ascorbic acid
BLM: Bleomycin
CAMF: Cancer-associated myofibroblasts
DEGs: Differentially expressed genes
DMOG: Dimethyloxalylglycine
GE: Gene ontology
HIF-1: Hypoxia-inducible factor 1
HIF-1α-ac: HIF-1α activator
HIF-1α-i: HIF-1α inhibitor
IP: Interstitial pneumonia
KEGG: Kyoto Encyclopedia of Genes and Genomes
LC: Lung cancer
LLC: Lewis lung carcinoma
NSCLC: Non-small cell lung cancer
OA: Oropharyngeal aspiration
PCA: Principal component analysis
PC-1: Principal component-1
RT-qPCR: Reverse transcription-quantitative PCR
TAM: Tumor-associated macrophages
TIMP-1: Tissue inhibitor of metalloproteinase-1
Treg: Regulatory T cell
VEGFA: Vascular endothelial growth factor A
