# Supplementary material for: Hypoxia-inducible factor 1α modulates interstitial pneumonia-mediated lung cancer progression
Source: J Transl Med. 2023 Nov 27;21:857. doi: 10.1186/s12967-023-04756-6 (PMC10680219; doi:10.1186/s12967-023-04756-6)
Supplement: Supplementary file 1 — Additional file 1: Fig. S1. Comparison of gene expression in each model. Fig. S2. Comparison of Hif1a expression between the sorted cancer cells from the IP+LC and LC models. Fig. S3. Comparison of side effects in each group of Fig. 3. Fig. S4. Effect of AsA on lung cancer alone model. Fig. S5. Effect of AsA on IP alone model. Fig. S6. Comparison of side effects in each group of Fig. 4. Fig. S7. Effect of AsA on lung cancer with interstitial pneumonia (IP). Fig. S8. Interstitial pneumonia (IP)-mediated changes in the tumor microenvironment based on flow cytometry. Fig. S9. Comparison of side effects in each group of Fig. 6. Fig. S10. Prognosis of patients with non-small cell lung cancer with and without interstitial pneumonia (IP). [file 12967_2023_4756_MOESM1_ESM.docx]

**Additional file 1**

**Hypoxia-inducible factor 1α modulates interstitial pneumonia-mediated lung cancer progression**

Kiyofumi Shimoji^1^, Taku Nakashima^1^*, Takeshi Masuda^1^, Masashi Namba^1^, Shinjiro Sakamoto^1^, Kakuhiro Yamaguchi^1^, Yasushi Horimasu^1^, Takahiro Mimae^2^, Shintaro Miyamoto^1^, Hiroshi Iwamoto^1^, Kazunori Fujitaka^1^, Hironobu Hamada^3^, Morihito Okada^2^, Noboru Hattori^1^

The Additional file 1 includes 10 Figures.

**Fig. S1. Comparison of gene expressions in each model.**

(A) Experimental scheme of the development of the IP model. O.A., oropharyngeal administration. BLM, bleomycin. HC, Healthy control. (B–E) Comparison of (B) *Hif1a*, (C) *Timp1*, (D) *Vegfa* and (E) *Col1a1* between the HC, IP model, LC model and IP+LC model on day 21 (n = 4–5/group). *P < 0.05.

**Fig. S2. Comparison of *Hif1a* expression between the sorted cancer cells of the IP+LC and LC models.**

*P < 0.05.

**Fig. S3. Comparison of side effects in each group of Fig. 3.**

(A–E) Comparison of (A) body weight change during the experimental period, (B) red blood cells; RBC, (C) hemoglobin; Hb, (D) white blood cells; WBC and (D) platelet; PLT between the Control, HIF-1α-i, AsA+, HC, HC (HIF-1α-i) and HC (AsA) groups on day 28 (n=5-8/group). AsA, ascorbic acid. HIF-1α, hypoxia-inducible factor 1α. HIF-1α-i, HIF-1α inhibitor. HC, Healthy control. NS represents not significant. **P* < 0.05.

**Fig. S4. Effect of AsA on lung cancer alone model.**

(A) Experimental evaluation scheme of AsA treatment in the LC model. AsA, ascorbic acid. i.p, intraperitoneal injection. (B) Hematoxylin and eosin staining of lung sections and macroscopic evaluation of the LC and AsA+LC groups on day 14. The results are shown for LLC. Scale bar: 300 μm. (C and D) Comparison of (C) tumor volume and (D) weight of mediastinal lymph nodes between the LC and AsA+LC groups on day 14 (n = 7/group). NS represents not significant. *P < 0.05.

**Fig. S5. Effect of AsA on IP alone model.**

(A) Experimental evaluation scheme of AsA treatment in the IP model using C57BL/6 mice. AsA, ascorbic acid. BLM, bleomycin. O.A., oropharyngeal administration. i.p, intraperitoneal injection. (B) Comparison of hydroxyproline levels among the sham, IP, and AsA+IP groups on day 28 (n = 5–7/group). *P < 0.05, **P < 0.01. (C) Hematoxylin, eosin, and Masson’s trichrome staining of lung tissue sections obtained from the sham, IP, and AsA+IP groups on day 28. Scale bar: 300 μm.

**Fig. S6. Comparison of side effects in each group of Fig. 4.**

(A–E) Comparison of (A) body weight change during the experimental period, (B) red blood cells; RBC, (C) hemoglobin; Hb, (D) white blood cells; WBC and (D) platelet; PLT between the LC, IP+LC, AsA+IP+LC, HC and HC (AsA) groups on day 28 (n=5-8/group). AsA, ascorbic acid. HC, Healthy control. NS represents not significant. *P < 0.05.

**Fig. S7. Effect of AsA on lung cancer with interstitial pneumonia (IP).**

Immunohistochemical staining of KLN205 tumor tissue from the LC, IP+LC, and AsA+ IP+LC groups with anti-HIF-1α, anti-TIMP-1 and anti-VEGFA antibodies on day 28. HIF-1α, hypoxia-inducible factor 1α. TIMP-1, tissue inhibitor of metalloproteinase-1. VEGFA, vascular endothelial growth factor A. Scale bar: 50 μm.

**Fig. S8. Interstitial pneumonia (IP)-mediated changes in the tumor microenvironment based on flow cytometry.**

(A–F) Flow cytometry for examining cancer and tumor microenvironment in the LC, IP+LC, and AsA+IP+LC groups on day 28. For injected cancer cells, KLN205 was used. AsA, ascorbic acid. Comparison of (A) cancer cell fraction, (B) tumor-associated macrophages (TAM) fraction, (C) cancer-associated myofibroblasts (CAMF) fraction, (D) regulatory T cells (Treg) fraction, (E) cytotoxic T lymphocytes (CTL) fraction, and (F) CTL / Treg ratio between the LC, IP+LC, and AsA+IP+LC groups on day 28 (n = 6/group). NS represents not significant. *P < 0.05, **P < 0.01. (G) Correlations between HIF-1α expression in tumor tissue and cancer cell fraction, TAM fraction, CAMF fraction, and CTL / Treg ratio on day 28. HIF-1α, hypoxia-inducible factor 1.

**Fig. S9. Comparison of side effects in each group of Fig. 6.**

(A–E) Comparison of (A) body weight change during the experimental period, (B) red blood cells; RBC, (C) hemoglobin; Hb, (D) white blood cells; WBC and (D) platelet; PLT between the AsA–, AsA+, AsA+HIF-1α-ac, HC and HC (AsA) groups on day 28 (n=5-8/group). AsA, ascorbic acid. HIF-1α, hypoxia-inducible factor 1α. HIF-1α-ac, HIF-1α activator. HC, Healthy control. NS represents not significant.

**Fig. S10. Prognosis of patients with non-small cell lung cancer with and without interstitial pneumonia (IP).**

(A) Kaplan–Meier survival curve for overall survival period (OS) between the IP (+) and IP (–) groups in cohort 1. (B) HIF-1α and Ki-67 expression in tumor specimens of the IP (+) and IP (–) groups in cohort 1. Representative images of tumor specimens stained with an antibody against human HIF-1α or Ki-67 via immunohistochemistry. (C) Comparison of Ki-67 expression in the tumor area of the resected lung between the IP (+) and IP (–) groups in cohort 1 (n = 20–21/group). *P < 0.05. (D) Kaplan–Meier survival curve for OS between the IP (+) and IP (–) groups in cohort 2.
